# The lifestyle factors of medical doctors in academic hospitals, Bloemfontein, Free State

**DOI:** 10.4102/safp.v66i1.5979

**Published:** 2024-10-30

**Authors:** Deepa C. Alexander, Leané Lessing, Huibré Botes, Fredré Conradie, Lu-Zahn Jansen van Rensburg, Karien Nel, Emmarentia Pienaar, Maryke Prinsloo, Lialma Sinclair, Cornel van Rooyen

**Affiliations:** 1 Department of Paediatrics and Child Health, Faculty of Health Sciences, University of the Free State, Bloemfontein, South Africa; 2 Department of Biostatistics, Faculty of Health Sciences, University of the Free State, Bloemfontein, South Africa

**Keywords:** dietary and lifestyle, medical doctors, academic, Bloemfontein, standards of health

## Abstract

**Background:**

Lifestyle factors of medical doctors are essential to their health and well-being. Previous studies omitted factors that constituted a healthy lifestyle and did not differentiate between various medical specialties or level of seniority which may expose doctors to different stress levels, workload and responsibility. The study assessed the lifestyle factors of medical doctors and compared them between departments, levels of seniority, years of experience and gender according to globally recognised health standards.

**Methods:**

This descriptive cross-sectional study collected data using a questionnaire created by the researchers based on healthy lifestyle factors and was administered online. Access was given to all doctors from four large departments, employed at two academic hospitals in Bloemfontein, Free State who agreed to participate in the study.

**Results:**

Consultants from paediatrics, with 6+ years in medical practice, had the healthiest lifestyles. Registrars and interns from surgical disciplines such as obstetrics and gynaecology and surgery, with 1–5 years of medical practice, showed unhealthy lifestyles with inadequate sleep and exercise.

**Conclusion:**

The challenge remains how medical doctors can live a healthy lifestyle while managing a demanding schedule. This may impact on the management of their patients and the doctors’ overall health and well-being. We recommend improving the working conditions by providing healthy meals on-site at hospitals with adequate breaks, reducing the heavy workload and providing exercise facilities.

**Contribution:**

The findings from this article may help improve the lifestyles of the identified groups of at-risk doctors and assist them in seeking ways to improve upon this.

## Introduction

The World Health Organization (WHO) defines health as ‘complete physical, mental and social well-being and not merely the absence of disease or infirmity’.^1^ In the United Kingdom, a study demonstrated that 44% of medical doctors felt that their health and well-being was negatively impacted because of their work, most of which ascribed this to stress from the heavy workload experienced and inability to maintain a healthy work-life balance.^2^

Poor lifestyle choices such as tobacco use, excessive alcohol consumption and inadequate physical activity directly impacts health and well-being, which may lead to chronic diseases according to the Center for Disease Control and Prevention (CDC).^3^ In 2021, a study revealed that 16.3% of adult South Africans were diagnosed with single or multiple chronic diseases,^4^ such as heart disease, diabetes and cancer.^5^ Medical professionals should therefore be aware of their health status compared to globally recognised standards to prevent the development of chronic diseases.^6^

With regard to healthy eating habits, a study conducted in Pakistan revealed that only 1 in every 1190 healthcare professionals was eating a diet according to the United States Department of Agriculture Dietary Guidelines; others ate more foods from the protein group and less from the fruits, vegetables and dairy groups.^7^ In South Africa, a study conducted in a district hospital in KwaZulu-Natal, showed that most healthcare professionals do not follow healthy diets.^8^ These findings suggest that scientific-based dietary guidelines are not adhered to.

A study performed in Cape Town, South Africa, looked at nurses’ lifestyle behaviours, health priorities and barriers to a healthy lifestyle. The findings suggest that the hospital environment was perceived to negatively influence the nurses’ lifestyle because of the long working hours and the availability of predominantly unhealthy food choices at the available food service.^9^ This highlights the need for better dietary options for healthcare professionals to maintain their health.

Burnout has been reported by medical doctors because of high levels of stress, which can result in poor decision making, hostile attitude towards patients and co-workers, and avoidable medical errors.^10^ There have been reports on the effectiveness of strategies against burnout. Examples include participation in group discussions, acknowledging work stressors and providing expert advice and encouragement of self-care such as getting enough exercise, rest and spending time with loved ones to maintain a positive attitude and balanced life.^10^ Another study mentioned potential strategies for preventing burnout, which include improving awareness, promoting mindfulness, availability of mental health service, using digital technologies and organisational approaches to creating an enabling environment.^11^

To date there have been no comparative studies that differentiate between the lifestyle factors of different medical specialities, level of seniority, and the various ages of the consultants, registrars and interns. Consultants are senior doctors who have specialised in an area of medicine.^12^ Registrars have completed their foundation but are busy with specialty training.^12^ Interns are in their first 2 years of post-medical school training, cannot practice unsupervised and are therefore confined to the training programme they are enrolled in.^12^ This differentiation is vital as consultants, registrars and interns are exposed to different levels of stress, workload, responsibility and academic studies, and are required to deal with various scenarios where patients are in life-threatening situations.

The study aimed to assess the lifestyle factors of medical doctors in four large departments in two academic teaching hospitals in Bloemfontein, Free State.

The secondary objectives were to:

- Compare lifestyle factors (sleep, dietary habits, physical exercise, stress, water drinking habits and smoking habits) of doctors between different departments, levels of seniority, years of medical practice and gender.
- Compare lifestyle factors of doctors to globally recognised standards.

## Research methods and design

### Study design and sampling

A descriptive cross-sectional study design was used. The target population included medical doctors from four large departments, who were employed full-time at the Universitas Academic Hospital and Pelonomi Tertiary Hospital, Bloemfontein, Free State. The selection of the population was purposive and non-randomised to 230 doctors. Those who met specific criteria and were willing to participate by voluntarily consenting to the study were included in the sample, which totalled 73 doctors. These were interns, consultants and registrars from the following four disciplines: obstetrics and gynaecology, internal medicine, paediatrics and surgery. Specific departments were selected as they were among the biggest and most representative with similar work hours, environment and staff establishment. Medical officers were excluded as there are few on the staff establishment and some are not employed full-time whereas some are on a contract basis. Those who were not willing to participate, or those who were on leave at the time of the survey were not included in the sample.

### Data collection and measurement

The online questionnaire was designed by the researchers according to the literature found regarding recommendations for a healthy lifestyle. Two to three rounds of questionnaires and information documents were distributed to the various departments’ staff members via email and mobile phone numbers along with reminders. A quick-response (QR) code was created and added to posters that were put up at the various departments.

Respondents had to indicate their department, level of seniority, years of medical practice, gender and marital status. Information on height and weight (to determine body mass index [BMI]) were also collected. Key lifestyle factors included in the questionnaire were hours of sleep per night, dietary habits such as foods consumed (fruits, vegetables, coffee, salt, sugary snacks) per week and length of lunch break, minutes of physical exercise performed per week, daily stress level experienced scaled from 1 (lowest) to 4 (highest) and physical, emotional and behavioural changes experienced, water consumption per day and smoking habits.

In an open-ended question, the respondents were asked to give recommendations regarding their own experience to help doctors pursue a healthier lifestyle regarding exercise, stress, sleep, smoking and diet.

### Pilot study

A pilot study was conducted with 12 consultants from the Department of Paediatrics at the University of the Free State (UFS) to test the understanding and validity of the questionnaire. Questionnaire links were emailed to the respondents. After the pilot study, no changes were made to the questionnaire. The data collected from the pilot study were included in the data analysis.

### Data analysis

Data were analysed by the Department of Biostatistics, Faculty of Health Sciences, UFS, using SAS version 9.4 (SAS Institute Inc., Cary, North Carolina, United States). Continuous variables were summarised by medians, minimum, maximum or percentiles. Categorical variables were summarised by frequencies and percentages. Differences between groups were evaluated using appropriate statistical tests (Chi-square or Fisher’s exact test) for unpaired data. A *p*-value of ≤ 0.05 was considered statistically significant. Subgroups were compared using a 95% confidence interval (CI).

### Ethical considerations

The protocol was approved by the Health Sciences Research Ethics Committee (HSREC) of the Faculty of Health Sciences [UFS-HSD2020/0458/2909]. Permission to conduct the study was obtained from the Free State Department of Health and University of the Free State (UFS) School of Clinical Medicine. The head of the department for each speciality was informed of the study.

Because of the COVID-19 pandemic and nationwide lockdown, the survey had to be conducted online. An information document outlining the study was emailed to the population group, followed by a consent form, after which if the participant agreed and gave voluntary consent it proceeded to the online questionnaire. The respondents’ identities remained confidential. Data were saved on a computer and online drive. These files were password-protected and were only made known to the researchers in the group.

## Results

Of the 230 medical doctors who met the inclusion criteria, there were 61 respondents to the questionnaire who were included in the study. With the 12 participants from the pilot study, the total sample size was 73 doctors. (response rate 31.7%). As shown in Table 1, the highest response rate was from interns (46.6%) and from the Department of Paediatrics (43.5%).

**TABLE 1:** Distribution of respondents per department and level of seniority.

Department	Level of seniority						Total	
	Registrar		Consultant		Intern		Respondents out of total doctors (*n*)	Response rate (%)
	Respondents out of total registrars (*n*)	Response rate (%)	Respondents out of total consultants (*n*)	Response rate (%)	Respondents out of total interns (*n*)	Response rate (%)		
Internal Medicine	3/26	11.5	7/23	30.4	10/17	58.8	20/66	30.3
Obstetrics and Gynaecology	9/18	50.0	3/13	23.1	0/9	0.0	12/40	30.0
Paediatrics	7/27	25.9	13/28	46.4	10/14	71.4	30/69	43.5
Surgery	2/21	9.5	2/16	12.5	7/18	38.9	11/55	20.0
**Total**	**21/92**	**22.8**	**25/80**	**31.3**	**27/58**	**46.6**	**73/230**	**31.7**

Table 2 summarises the demographic work-related profile of the respondents. Over half (54.8%) of the respondents were male, 55.6% were married, and 37.5% had never been married. The highest percentage (39.7%) of respondents had been in practice for > 10 years. The department represented best was paediatrics (41.1%), and 37.0% of all the respondents were interns.

**TABLE 2:** Frequency table for demographic and work-related profile of respondents.

Variables	*n*	%
**Gender (*N* = 73)**		
Female	33	45.2
Male	40	54.8
**Marital status (*N* = 72)†**		
Currently married	40	55.6
Divorced	2	2.8
Living together	3	4.2
Never married	27	37.5
**Years of medical practice (*N* = 73)**		
1–5	28	38.4
6–10	16	21.9
> 10	29	39.7
**Level of seniority (*N* = 73)**		
Consultant	25	34.3
Intern	27	37.0
Registrar	21	26.5
**Department (*N* = 73)**		
Internal Medicine	20	27.4
Obstetrics and Gynaecology	12	16.4
Paediatrics	30	41.1
Surgery	11	15.1

† , Missing *n* = 1.

### Dietary and lifestyle factors

#### Dietary habits

The respondents ate fruit a median of 5 days per week (range 0–7 days). The majority indicated consuming 1–2 servings of fruit (83.1%) and 1–2 servings of vegetables (70.0%) per day. The respondents ate vegetables a median of 5 days per week (range 1–7 days).

The highest percentage (38.4%) of respondents rarely added extra salt or salty sauces to food before or during a meal. Although most respondents (57.5%) were unaware of the recommended daily sugar allowance, 61.1% indicated that they consumed less than the maximum according to health standard recommendations. Half (52.1%) of the respondents consumed 1–2 servings of sugary treats (chocolates, drinks, sweets) in a typical week.

#### Smoking and coffee and water consumption

Only 5.5% of the responding doctors reported that they currently smoke some form of tobacco. Respondents consumed a median of 4 cups of water per day. Half of the doctors (49.3%) reported drinking 1–2 cups of coffee per day, 24.7% drank 3–4 cups per day, and only 8.2% indicated that they drank 5–6 cups per day. The remaining 17.8% reported not drinking coffee at all.

#### Physical exercise and sleep

The highest percentage (32.9%) of respondents exercised 1–2 days per week, while 23.3% did not exercise at all. More than half (54.9%) spent less than 100 min per week on moderate-intensity exercise (e.g. brisk walking, general gardening). The highest percentage of respondents (42.3%) did not do any vigorous-intensity exercise in a typical week, while 38.0% indicated 75 min – 100 min of vigorous-intensity exercise (e.g. running, strength training, hiking uphill). The respondents slept a median of 6.75 h a night (range 4 h – 10 h).

#### Stress

According to the respondents, their daily level of stress, with level 1 being the lowest and level 4 being the highest, was 6.9% for level 1, 26.0% for level 2, 39.7% for level 3, and 27.4% for level 4. Table 3 summarises the respondents’ physical, emotional and behavioural changes because of stress.

**TABLE 3:** Questions on changes experienced because of stress (*n* = 73).†

Category	*n*	%
**Do you ever feel physical changes because of stress?**		
None	27	37.0
Headaches	37	50.7
Indigestion	16	21.9
Nausea	8	11.0
Rapid breathing	8	11.0
**Do you ever feel emotional changes because of stress?**		
None	7	9.6
Frustration	43	58.9
Anxiety	36	49.3
Depression	22	30.1
Anger	16	21.9
Fear	9	12.3
**Do you ever experience behavioural changes because of stress?**		
None	15	20.6
Irritability	41	56.2
Withdrawal	23	31.5
Tearful	15	20.6
Indecisiveness	13	17.8

† , multiple-response questions.

Some respondents indicated that they do not experience any physical (37.0%), emotional (9.6%) and/or behavioural (20.6%) changes because of stress. The two most cited physical changes were headaches (50.7%) and indigestion (21.9%). Emotional changes were frustration (58.9%) and anxiety (49.3%), while irritability (56.2%) and withdrawal (31.5%) were the two most cited behavioural changes.

All demographic variables were compared to all the dietary and lifestyle factors.

#### Department of the doctor

Statistically significant associations were found between the department of the doctor and sleep, BMI and dietary habits (Table 4).

**TABLE 4:** Department of doctor and lifestyle factors.

Variable	Department								*p*
	Internal Medicine		Obstetrics and Gynaecology		Paediatrics		Surgery		
	*n*	%	*n*	%	*n*	%	*n*	%	
**BMI**	-	-	-	-	-	-	-	-	0.0035
Normal weight (*n* = 36)	9	47.3	3	25.0	22	73.3	2	18.2	-
Overweight (*n* = 28)	8	42.1	8	66.7	7	23.3	5	45.5	-
Obese (*n* = 8)	2	10.5	1	8.3	1	3.3	4	36.4	-
**Amount of sleep per night**	-	-	-	-	-	-	-	-	0.0019
< 7 h sleep (*n* = 36)	8	40.0	12	100.0	11	36.7	5	50.0	-
> 7 h sleep (*n* = 36)	12	60.0	0	0	19	63.3	5	50.0	-
**Number of minutes for lunch per day**	-	-	-	-	-	-	-	-	0.0310
No lunch break (*n* = 21)	11	55.0	2	16.7	5	16.7	3	30.0	-
< 30 min (*n* = 35)	4	20.0	6	50.0	20	66.7	5	50.0	-
≥ 30 min (*n* = 16)	5	25.0	4	33.3	5	16.7	2	20.0	-

Note: Internal Medicine: BMI: *n* = 19†; Amount of sleep per night: *n* = 20; Number of minutes for lunch per day: *n* = 20. Obstetrics and Gynaecology: BMI: *n* = 12; Amount of sleep per night: *n* = 12; Number of minutes for lunch per day: *n* = 12. Paediatrics: BMI: *n* = 30; Amount of sleep per night: *n* = 30; Number of minutes for lunch per day: *n* = 30. Surgery: BMI: *n* = 11; Amount of sleep per night: *n* = 10†; Number of minutes for lunch per day: *n* = 10.

† , Missing *n* = 1.

According to their BMI, most (73.3%) respondents from paediatrics had a normal weight, while 66.7% of the respondents from obstetrics and gynaecology were overweight. All respondents from obstetrics and gynaecology slept < 7 h per night, while 63.3% of respondents from paediatrics slept > 7 h per night. More than half (55.0%) of the respondents from internal medicine reported not taking a lunch break, 66.7% of the respondents from paediatrics had a lunch break of < 30 min, while the highest percentage (33.3%) of respondents with a lunch break of > 30 min were from obstetrics and gynaecology.

#### The seniority of the doctor

Statistically significant associations between level of seniority and sleep, physical exercise, and levels of stress were found (Table 5).

**TABLE 5:** Level of seniority of doctor and lifestyle factors.

Variable	Level of seniority						*p*
	Consultant		Intern		Registrar		
	*n*	%	*n*	%	*n*	%	
**Frequency of exercise per week**	-	-	-	-	-	-	0.0004
≤ 2 days per week (*n* = 41)	7	28.0	16	59.3	18	85.7	-
≥ 3 days per week (*n* = 32)	18	72.0	11	40.7	3	14.3	-
**Amount of sleep per night**	-	-	-	-	-	-	0.0416
< 7 h sleep (*n* = 36)	12	48.0	9	34.6	15	71.4	-
> 7 h sleep (*n* = 36)	13	52.0	17	65.4	6	28.6	-
**Levels of stress per day**	-	-	-	-	-	-	0.0007
Low to medium stress levels (*n* = 24)	6	24.0	16	59.3	2	9.5	-
High to very high stress levels (*n* = 49)	19	76.0	11	40.7	19	90.5	-

Note: Consultant: Frequency of exercise per week: *n* = 25; Amount of sleep per night: *n* = 25; Levels of stress per day: *n* = 25. Intern: Frequency of exercise per week: *n* = 27; Amount of sleep per night: *n* = 26†; Levels of stress per day: *n* = 27. Registrar: Frequency of exercise per week: *n* = 21; Amount of sleep per night: *n* = 21; Levels of stress per day: *n* = 21.

† , Missing *n* = 1.

Majority of the registrars (85.7%) exercised ≤ 2 days per week, while 72.0% of the consultants exercised ≥ 3 days per week. Most (71.4%) registrars slept < 7 h per night, while consultants (52.0%) and interns (65.4%) slept > 7 h per night. Most individuals who reported low to medium stress levels were interns (59.3%), while most registrars (90.5%) and consultants (76%) reported that they experienced high to very high stress levels.

#### Years in medical practice

There was a statistically significant association between years in medical practice and dietary habits (Table 6).

**TABLE 6:** Years in medical practice of doctor and lifestyle factors.

Variable	Years of medical practice						*p*
	1–5 years (*n* = 27)†		6–10 years (*n* = 16)		> 10 years (*n* = 29)		
	*n*	%	*n*	%	*n*	%	
**Number of minutes for lunch per day**	-	-	-	-	-	-	0.0376
No lunch break (*n* = 21)	12	44.4	5	31.3	4	13.8	-
< 30 min (*n* = 35)	13	48.2	6	37.5	16	55.2	-
≥ 30 min (*n* = 16)	2	7.4	5	31.3	9	31.0	-

† , Missing *n* = 1.

The highest percentage (44.4%) of respondents who did not take lunch breaks had 1–5 years of medical practice. Over half (55.2%) of the respondents with > 10 years of medical practice took lunch breaks < 30 min.

#### Marital status and stress levels

Statistically significant associations were found for marital status and stress levels (rated 1–4, where 4 is the highest) (Table 7). Being married led to higher levels of stress, 74.4% experienced level 3–4 stress, whereas 82.8% of the unmarried had experienced level 2–3 stress.

**TABLE 7:** Marital status and levels of stress categories (*n* = 72).

Variable	Marital status				*p*
	Married (*n* = 43)		Unmarried (*n* = 29)		
	*n*	%	*n*	%
**Stress levels**	-	-	-	-	0.0469
1 (lowest) (*n* = 5)	3	7.0	2	6.9	-
2 (*n* = 19)	8	18.6	11	38.0	-
3 (*n* = 29)	16	37.2	13	44.8	-
4 (highest) (*n* = 19)	16	37.2	3	10.3	-

#### Gender and stress symptoms

There were also statistically significant associations between gender and stress symptoms. Seven (21.2%) female doctors experienced nausea as a physical symptom (*p* = 0.0195), whereas 50.0% of the male doctors reported indigestion (*p* = 0.0151). A behavioural change of irritability was noticed in 36.4% of the females (*p* = 0.0024).

Recommendations to improve diet and lifestyle factors are shown in Table 8. Of the 38 responses, 31.5% recommended the provision of affordable, healthy and convenient food at the hospitals.

**TABLE 8:** Recommendations to improve the lifestyle factors of doctors (*n* = 38).

Suggested action to improve diet and lifestyle of doctors	*n*	%
Provision of affordable healthy convenient food at hospitals	12	31.5
Reduced workload by employing more doctors	8	21.0
Scheduled specific lunch breaks	5	13.2
Provide exercise facilities at hospital	5	13.2
Healthy living campaigns among doctors via messages on their mobile phones	4	10.5
Workshops on coping with stress	2	5.3
Other (sleep more and plan your day)	2	5.3

## Discussion

This study assessed the lifestyle factors of interns, registrars and consultant medical doctors in two academic hospitals from four large departments and compared the findings to globally recognised standards of health. Comparisons were drawn between the demographic variables and these factors. There were 73 respondents in total, of which over half (54.8%) were male, 55.6% were married at the time of the study, and just over a third (39.7%) had more than 10 years of work experience. The highest response rate was from interns (46.6%) and from the Department of Paediatrics (43.5%).

According to the South African Food based dietary guideline from 2013, living a healthy lifestyle includes eating from a variety of food groups such as fruits and vegetables, starchy foods, beans and lentils, dairy products and lean meats. They also recommend being active and to drink plenty of water, while consuming fats, sugary foods and salt sparingly.^13^

### The profile of lifestyle factors observed in this study

With regard to the number of suggested fruits and vegetables to consume, the Dietary Guidelines for Americans^14^ recommend a daily intake of 2 to 2.5 cups of vegetables and 1.5 to 2 cups of fruit for adults. A review article mentioned that the intake of fruit and vegetables in many countries, especially developing ones, is inadequate.^15^ The sample of doctors in our study did not consume the recommended daily amount of fruit and vegetables but rather only a median of five times per week.

The recommended maximum sugar allowance per day, according to the American Heart Association is 6 or less teaspoons in females and 9 or less teaspoons in males.^16^ Our study found that 61.1% of respondents are within the maximum limit of sugar intake per day but half (52.1%) ate 1–2 servings of sugary treats (chocolates, drinks and sweets) in a week. Reviewing the water and coffee consumption, our doctors drank a median of four cups of water per day, less than the recommended 4–6 cups per day.^17^ Three to five cups of coffee daily is still considered healthy, but the maximum caffeine intake is 400 mg per day.^18^ According to our results, most doctors are within the healthy range of daily coffee consumption which is similar to study conducted in Europe which also found that the doctors in their study population stayed within the recommended range.^19^

The average amount of sleep needed is between 7 h and 9 h for an adult per night.^20^ In a study conducted on urologists across Europe,^19^ more than 60% slept fewer than 6 h per night, similar to our findings where half the doctors slept less than the recommended minimum. According to a study based on a joint consensus statement of the American Academy of Sleep Medicine and Sleep Research Society,^21^ adults should regularly sleep 7 h or more hours per night to promote optimal health whereas, sleeping less can lead to health problems such as weight gain, heart disease and stroke, hypertension, mental depression, impaired immune function, impaired performance and a greater risk of accidents and errors.^21^

Good habits such as physical activity assists one in maintaining a healthy weight and preventing chronic diseases.^6^ Even though the health benefits are greater with higher-intensity exercise, just small increases in daily physical activity can improve overall health.^3^ A study of the lifestyles of doctors in Ireland found that most were compliant with exercising the minimal required amount but junior doctors performed less exercise overall compared to their seniors.^22^ In our study, a fair amount (23.3%) of participants did not exercise at all, whereas only 38% performed the recommended 75 min - 100 min of vigorous-intensity exercise per week.^23^

It has been mentioned that too little sleep and exercise can possibly contribute to stress and burnout among doctors.^3^ Buchwald and Hobfoll stated that burnout occurs if the individual perceives that their personal and work resources have been depleted and they are unable to replenish them.^24^ A study by Kumar et al. highlighted the importance of addressing burnout among doctors to improve patient outcomes and the well-being of healthcare providers.^10^ With regard to levels of stress experienced, the registrar group reported the highest. The two most cited physical changes in response to stress were headaches and indigestion with emotional changes being frustration and anxiety. Behavioural changes reported were irritability and withdrawal.

With regard to poor lifestyle choices, our study found that only 5.5% of doctors were smoking some form of tobacco. This corresponds to a similar study that found that a small minority (9.0%) of doctors smoke.^22^

### Comparisons of the demographic profile to the lifestyle factors between various groups of doctors

We wanted to assess whether certain departments had a difference between their lifestyle choices. Regarding hours of sleep, our study showed that doctors from surgical disciplines had poor quantity of sleep. All respondents from the Department of Obstetrics and Gynaecology and half from the Department of Surgery slept < 7 h per night, whereas, about two-thirds of paediatrics and internal medicine slept the recommended 7 h – 9 h per night. A study among Nigerian doctors found that the entire study population were poor sleepers with prolonged work hours and heightened daytime sleepiness.^25^

With regard to healthy weight, the United States Physicians’ Health Study revealed that 44.0% of male physicians are overweight, with 6.0% being classified as obese.^26^ It was found that the doctors from the Department of Obstetrics and Gynaecology took the most extended lunch breaks and 66.7% were overweight, whereas more than half (55.0%) of internal medicine doctors reported not taking lunch breaks at all and 47.3% of them were within a normal weight category. More than one-third (36.4%) of doctors from the Department of Surgery were obese. And the healthiest doctors were from the Department of Paediatrics reporting 73.3% normal BMI and internal medicine of whom 47.3% had the healthiest lifestyle habits.

Comparing lifestyles between different levels of seniority, we found a higher percentage of registrars had poorer lifestyles with regard to sleep, exercise and stress. Most registrars had a shorter sleep duration per night in comparison to consultants and interns. In South Africa, residents are comparable to registrars and consultants to attendings.^12^ A study among residents and attending doctors in the United States found that attending doctors had slightly better sleep quality and longer sleep duration than residents.^27^

Most registrars exercised less frequently per week in comparison to consultants and they (90.5%) reported experiencing high to very high stress levels. A study performed in the United States found that residents suffering from burnout had higher levels of depression and perceived stress with lower levels of self-efficacy.^28^ Residents are undergoing additional clinical training to become specialists and typically work in hospitals and clinics while being supervised by attending doctors.^29^

Regarding experience and years in medical practice, most respondents who had 1–5 years of medical practice did not take lunch breaks and those who did had >10 years work experience.

With regard to gender, changes experienced because of stress were reported to be different among male and female respondents. Physical changes reported by male doctors were indigestion as the main symptom whereas in females it was nausea of which more than one-third also exhibited behavioural changes such as irritability. Doctors who were married experienced higher levels of stress compared to single or unmarried doctors who experienced low to moderate stress levels.

### Study’s limitations

Because of the coronavirus disease 2019 (COVID-19) restriction, the authors could not self-administer the questionnaires, leading to a lower response rate. More respondents could have enabled the researchers to use more intermediate and advanced statistical analyses, which could have avoided under- and over-representing specific departments and categories of doctors. Lastly, given the cross-sectional design of the study, the correlations found do not imply causality.

### Recommendations

If an intervention is planned to improve the lifestyle and dietary factors of doctors in Bloemfontein’s academic hospitals, the priority group will have to be registrars, and interns working in the Departments of Obstetrics and Gynaecology and General Surgery. Most consultants reported acceptable to good lifestyles and dietary habits.

The authors hope that the study’s findings will assist in health promotion among doctors working in academic hospitals and recommend a review of their working conditions to provide a sustainably healthy environment with healthy food and exercise programmes. This would produce doctors who can promote healthy living among their patients and become role models on living a healthy lifestyle while managing a demanding schedule.^30^

## Conclusion

This study has shown that the medical doctors in our sample population of academic hospitals in Bloemfontein do not follow recommended guidelines for preserving their health. Results show that although there were no excessive unhealthy habits of consuming too much sugar, salt and caffeine or tobacco smoking, they did eat a diet restricted of fruits, vegetables and water. Junior doctors do not sleep adequately and do not perform quality exercise, which may lead to burnout and stress. Regarding the healthiest lifestyle practices, consultants from the Paediatrics Department with 6 years or more experience in medical practice performed the best. In contrast, the typical profile of the doctors with the poorest lifestyle would be registrars and interns from the Departments of Obstetrics and Gynaecology and General Surgery with 1–5 years of medical practice.

The study also highlights the physical, emotional and behavioural changes because of stress most often experienced by doctors. This may be useful to screen symptoms of stress in doctors. Although senior doctors experience moderate stress, they have adequate sleep and exercise. However, this is lacking in registrars and may explain the higher levels of stress reported.

Recommendations made by the respondents to improve the diet and lifestyle of doctors in our setting show that the lack of lunch breaks, non-availability of healthy food at the hospital, a heavy workload, and no exercise facilities were the four key issues that could be improved upon.

Future studies are required to investigate lifestyle factors in medical doctors employed in other disciplines and to include more participants.
